# Esthesioneuroblastoma is not a member of the primitive peripheral neuroectodermal tumour-Ewing’s group

**DOI:** 10.1038/sj.bjc.6690734

**Published:** 1999-10

**Authors:** A Mezzelani, S Tornielli, F Minoletti, M A Pierotti, G Sozzi, S Pilotti

**Affiliations:** Division of Pathology and Cytology, Division of Experimental Oncology A, Istituto Nazionale per lo Studio e la Cura dei Tumori, Via G Venezian 1, Milan, 20133, Italy

**Keywords:** esthesioneuroblastoma, pPNETs-ETs, molecular analysis, distinct entity

## Abstract

Esthesioneuroblastoma (ENB) is a rare, site-specific, locally aggressive neuronal malignancy so far thought to belong to primitive peripheral neuroectodermal tumour-Ewing's tumour (pPNETs-ETs). Its anatomical location, in addition to morphologic, immunophenotypic and ultrastructural features, suggests its origin in the neuronal or neuroendocrine cells of the olfactory epithelium. However, the cytogenetic and molecular data currently available appear controversial on the presence of the typical translocation t(11;22)(q24;q12) and of trisomy 8, chromosomal changes that characterize the tumours belonging to the pPNETs-ETs. Herein we have analysed five ENB tumour specimens for trisomy 8 by fluorescence in situ hybridization (FISH), for the presence of EWS gene rearrangements by FISH, reverse transcription polymerase chain reaction and Southern blot analyses, as well as for the expression of the Ewing sarcoma-associated MIC2 antigen by immunohistochemistry. Neither EWS/FLI-I, EWS/ERG and EWS/FEV fusion genes nor MIC2 expression were found in any tumour, whereas trisomy 8 was found in one case only. Moreover, DNA from three cases analysed by Southern blot did not show EWS gene rearrangements. Our results support the evidence that ENB is not a member of the pPNETs-ETs. © 1999 Cancer Research Campaign


*British Journal of Cancer* (1999) **81**(4), 586–591
© 1999 Cancer Research Campaign
Article no. bjoc.1999.0734

### Esthesioneuroblastoma is not a member of the primitive peripheral neuroectodermal tumour-Ewing's group

**A Mezzelani[1]*, S Tornielli[2]*, F Minoletti[2], MA Pierotti[2], G Sozzi[2] and S Pilotti[1]**

[1]Division of Pathology and Cytology and [2]Division of Experimental Oncology A, Istituto Nazionale per lo Studio e la Cura dei Tumori, Via G Venezian, 1, 20133 Milan, Italy

**Summary** Esthesioneuroblastoma (ENB) is a rare, site-specific, locally aggressive neuronal malignancy so far thought to belong to primitive peripheral neuroectodermal tumour-Ewing's tumour (pPNETs-ETs). Its anatomical location, in addition to morphologic, immunophenotypic and ultrastructural features, suggests its origin in the neuronal or neuroendocrine cells of the olfactory epithelium. However, the cytogenetic and molecular data currently available appear controversial on the presence of the typical translocation t(11;22)(q24;q12) and of trisomy 8, chromosomal changes that characterize the tumours belonging to the pPNETs-ETs. Herein we have analysed five ENB tumour specimens for trisomy 8 by fluorescence in situ hybridization (FISH), for the presence of EWS gene rearrangements by FISH, reverse transcription polymerase chain reaction and Southern blot analyses, as well as for the expression of the Ewing sarcoma-associated MIC2 antigen by immunohistochemistry. Neither EWS/FLI-I, EWS/ERG and EWS/FEV fusion genes nor MIC2 expression were found in any tumour, whereas trisomy 8 was found in one case only. Moreover, DNA from three cases analysed by Southern blot did not show EWS gene rearrangements. Our results support the evidence that ENB is not a member of the pPNETs-ETs. © 1999 Cancer Research Campaign

**Keywords:** esthesioneuroblastoma; pPNETs-ETs; molecular analysis; distinct entity

Esthesioneuroblastoma (ENB) is a rare malignant tumour thought to derive from neuroendocrine cells of the olfactory epithelium on the basis of location, morphologic, immunophenotypic and ultrastructural features. In fact, well differentiated ENB is characterized by the presence of neuropil, Homer Wright and olfactory rosettes (Hyams et al, 1988), by a positive immunoreactivity for neuron-specific enolase, synaptophysin, chromogranin and neurofilaments and, ultrastructurally, by cytoplasmic processes, microtubules and dense core granules, all features consistent with an origin in the olfactory epithelium (Shanmugaratnam, 1991; Banerjee et al, 1992).

The neuroectodermal origin of ENB has been subsequently strengthened by cytogenetic and molecular findings. The presence of t(11;22)(q24;q12) translocation, the chromosome hallmark of Ewing's sarcoma, in addition to the single case of mesenchymal chondrosarcoma, was detected repeatedly in three other tumours: peripheral neuroepithelioma, Askin tumour and ENB (Heim et al, 1995). The presence of a common cytogenetic marker strengthened the hypothesis that all these four tumours are developmentally related and that they represent phenotypic variations of the same pathogenetic theme; for this reason they are defined as primitive peripheral neuroectodermal tumours, Ewing's tumours (pPNETs-ETs). In ENB the t(11;22)(q24;q12) has been detected in two out three metastatic cell lines (Whang-Peng et al, 1987; Cavazzana et al, 1988) and the presence of trisomy 8, a second non-random chromosomal aberration in pPNET-ETs, has been found in one of three short-term cultures of primary ENB (VanDevanter et al, 1991). The cytogenetic results were further confirmed by the molecular analysis carried out by reverse transcription polymerase chain reaction (RT-PCR) on six primary ENBs (Sorensen et al, 1996). In four of them (two of which originated in the previously cited cell lines) (Cavazzana et al, 1988), the presence of EWS/FLI-1 fusion transcript (Sorensen et al, 1994) was identified.

However, these cytogenetic-molecular-based data have been recently challenged by the results of two other studies. In the first study (Nelson et al, 1995,) 18 out of 18 ENBs resulted non-immunoreactively to the 12E7 monoclonal antibody, specific for the protein product of the MIC2 gene and known to reliably stain pPNETs-ETs, although not specifically (Chan et al, 1995). In the second study, 20 out of 20 ENBs were immunocytochemically MIC2-negative, and 11 of them were also EWS/FLI-1-negative for the presence of fusion transcripts. Furthermore one case, analysed by Southern blotting, did not show any EWS rearrangement (Argani et al, 1998).

To address the point of whether ENB shares common markers with pPNETs-ETs, we analysed five frozen samples of ENB, by RT-PCR, dual colour fluorescence in situ hybridization (FISH) and Southern blot. RT-PCR was performed to verify the occurrence of the EWS/FLI-1 fusion transcript, of the EWS/ERG by RNA originated from the less frequent t(21;22)(q22;q12) translocation reported in pPNETs-ETs (Sorensen et al, 1994), and of EWS/FEV transcript related to the very rare variant t(2;22)(q33;q12) (Peter et al, 1997). FISH on interphase nuclei was carried out in three cases using cosmid probes flanking the fusion gene (EWS/FLI-1) (Delattre et al, 1992; Zucman et al, 1992) and with a centromeric probe for chromosome 8 (D8Z2) in order to determine the presence of the t(11;22)(q24;12) translocation (Desmaze et al, 1994) and trisomy 8 respectively. Moreover, Southern blot analysis, with a cDNA probe spanning the breakpoint cluster region of EWS,

*Received 4 January 1999*
*Revised 13 April 1999*
*Accepted 22 April 1999*

*Correspondence to:* S Pilotti

*The authors contributed equally to the work.

**Table 1** Clinico-pathological features of the five ENBs

| Case | Age/Sex | Site | Sampling | Grading (WHO) | Staging (Kadish) | Immunostaining: Neuroendocrine markers/structural antigens NSE | Ch. | Syn. | S-100p | CKs | NFs | MIC2 |
|---|---|---|---|---|---|---|---|---|---|---|---|---|
| 1 | 35/F | Ethmoid | Complete surgical resection | 2 | A | +++ | +++ | +++ | + | – | + | – |
| 2 | 70/M | Ethmoid, maxillary sinus | Incomplete surgical resection | 3 | B | +++ | +++ | +++ | + | – | – | – |
| 3 | 50/F | Ethmoid, maxillary sinus | Incomplete surgical resection | 3 | C | +++ | +++ | +++ | + | – | – | – |
| 4 | 22/M | Ethmoid | Wide biopsy | 3 | C | +++ | – | + | + | + | – | – |
| 5 | 37/F | Nasal cavity | Wide biopsy | 4 | C | +++ | +++ | + | + | – | – | – |

Score applied to chromogranin, synaptophysin: positive cells < 20%: +; > 90%: +++. For neuron specific enolase, S-100 protein, cytocheratin and neurofilaments, see text.

was carried out in three cases in order to verify the integrity of the EWS gene.

None of the three different molecular approaches disclosed the presence of any fusion transcripts or EWS rearrangements, neither did immunohistochemistry detect any MIC2 positivity.

### MATERIALS AND METHODS

### Patients and tumours

The main clinico-pathologic features of the five patients under analysis are summarized in Table 1. Categorization and grading were performed according to the World Health Organization (WHO) classification (Shanmugaratnam, 1991), and pre-therapy staging according to the Kadish (1976) system.

Case 1 showed typical differentiated 'neuroblastoma like' growth pattern represented by nests of small round cells with reticular areas formed by tangles of neurites emanating from tumour cells. Areas with superimposable features were present in case II which in addition presented solid nests made up of larger tumour cells. The remaining three cases (III, V) were mainly made-up of well-defined cellular nests or lobules of variable size of cells with moderate pale eosinophilic cytoplasm with indistinct membranes surrounded by fibrovascular septae. In particular, a moderate amount of fibrillary background was present in case III and areas forming interconnecting lobules of tumour cells in case V.

All cases with fibrillary stroma (cases I, II, III) showed rosettes, whereas true olfactory (Flexner–Wintersteiner Wright) rosettes were present only in case IV.

### Immunocytochemistry

Immunophenotyping was carried out on formalin-fixed, paraffin-embedded material in all cases and in appropiate positive and negative controls using antibodies to the following antigens: neuron-specific enolase (NSE) (MIG-N3, Sanbio, 1:400 diluted), chromogranin (CRA237, Cambridge, 1:8000 diluted), synaptophysin (AO10, Dako, 1:100 diluted), S-100 protein (polyclonal, Dako, 1:4000 diluted), and cytocheratin and neurofilament (NF) cocktails as well as p30/32MIC2 (HBA71, 0.13, Labometric, 1/100 diluted; and CD99, Signet, 1:100 diluted). The applied cytocheratin (35β H11 Dako, and CAM 5.2 Becton-Dickinson, 1:100 and 1:20 diluted respectively) and neurofilament (RPN 1103, Amersham, and RPN 1104, Amersham, 1:33 and 1:50 diluted respectively) cocktails provided qualitative demonstration of human cytocheratin numbers 8 and 18, and neurofilament kDa 200 and 160 respectively. Antibody reactivity was visualized using the biotin–streptavidin method (Wood et al, 1981). For synaptophysin and cytocheratin antibodies, trypsin treatment of the sections was carried out. For NSE, chromogranin, NF antibodies and both HBA71 and CD99, heating-based antigen retrieval (Cattoretti et al, 1992) prior to the application of the primary antibody was applied.

Neuroendocrine markers and structural antigen immunophenotyping was consistent with the ENB diagnosis in all five cases (Table 1) (Frierson, 1990; Shanmugaratnam, 1991). Diffuse positivity for NSE and strong reactivity for chromogranin and/or synpatophysin was present in all five cases. Immunodecoration for S-100 protein and NFs was restricted to dendritic-subtentacular cells in five cases and neurophil formations in one. One case revealed focal staining for cytokeratins.

### Cytogenetic analysis

Metaphases obtained after the short-term (10 days) culture of case III were G-banded, and the karyotype of nine metaphases was determined.

### FISH on metaphases

Whole chromosome 22 painting (Cambio, Cambridge, UK) was hybridized, according to Pinkel et al (1986), on the metaphases from case III in order to confirm the cytogenetic results.

### FISH on interphase nuclei

*Probes*

F7 cosmid probes, located distal to the 22 chromosome breakpoint, and 1p3, which proximally flanks and overlaps half the breakpoint on chromosome 11 (Delattre et al, 1992; Zucman et al, 1992), were labelled by nick translation. Biotin-11-dUTP (Boehringer Mannheim, Germany) was incorporated in F7, and digoxigenin-11-dUTP (Boehringer Mannheim, Germany) in 1p3; 50 ng of both probes were then annealed to 5 μg of unlabelled human competitor DNA to suppress the repetitive sequences, and to 5 μg of carrier salmon sperm DNA. Botinylated chromosome 8 2-satellite (Oncor, Gaithersburg, MD, USA) was used for the detection of chromosome 8 trisomy according to the manufacturer's recommendations.

*© 1999 Cancer Research Campaign*

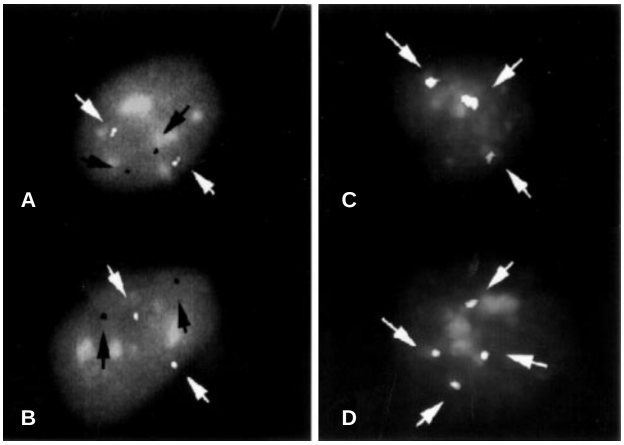

**Figure 1** Case III. (**A** and **B**) Two interphase nuclei showing the separated signals of F7 cosmid probes (white spots–white arrows), located distal to the 22 chromosome breakpoint, and 1p3 (black spots–black arrows), that proximally flanks and overlaps half the breakpoint on chromosome 11. When nuclei are in the G2 phase, the spots appear in pairs. (**C**) One interphase nucleus with three centromeric signals (white arrows) of chromosome 8. (**D**) One interphase nucleus with four centromeric signals (white arrows) of chromosome 8

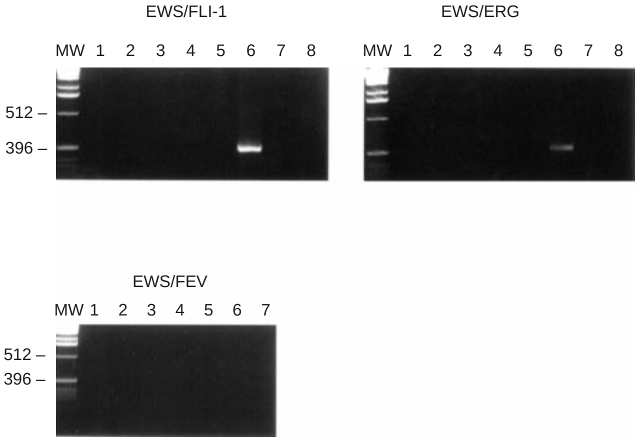

**Figure 2** RT-PCR results. MW: the molecular weight marker is 1 kb. EWS/FLI-1: samples 1–5 were tumour cDNA from cases I–V, sample 6 was tumour cDNA used as positive control for EWS/FLI-1 fusion transcript (500 bp), sample 7 was cDNA from normal tissue used as negative control, and sample 8 was the reaction mixture without cDNA; all were amplified by the EWS/FLI-1 primers 11.11 and 22.8. EWS/ERG: samples 1–8 are the same samples, except for the positive control that contains cDNA from EWS/ERG fusion transcript (490 bp), amplified by the EWS/ERG primers ERG11 and 22.8 EWS/FEV: samples 1–5 were tumour cDNA from cases I–V, sample 6 was the negative control and sample 7 was the reaction mixture without cDNA

### *FISH*

FISH was performed as previously described by Lichter and collaborators (1990) on cases I, III and IV. Independent experiments with a chromosome 8 centromeric probe and with F7 and 1p3 cosmid probes were performed respectively.

The hybridization signals of chromosome 8 2-satellite were detected by two layers of avidin–FITC (Vector, Burlingame, CA, USA). The signals of the F7 and 1p3 cosmids were simultaneously detected by two layers of avidin–FITC (which bound to the biotinylated F7 probe) and one layer of rhodamine-labelled anti-digoxigenin (Ab) followed by rabbit anti-sheep (Ab), and finally by rhodamine-labelled anti-rabbit (Ab) (Oncor, Gaithersburg, MD, USA) which bound to the digoxigenin of the 1p3 probe. Slides were then counterstained by DAPI (4,6,-diamidino-2-phenylindole dihydrochloride hydrate).

### *Digital signal detection*

Image acquisition was performed with a cooled CCD camera (Photometrics, Tucson, AZ, USA) coupled with a Zeiss fluorescence microscope and controlled by a Power Macintosh 7100/80. Frames of the nuclei were taken separately using the IPLab Spectrum (Signal Analytics) software package, and pseudo-coloured and merged using the Gene Join software (Ried et al, 1992).

### RT-PCR

A frozen tumour fragment from all cases as well as from two Ewing's tumours (ETs) with the EWS/FLI-1 and EWS/ERG fusion transcripts, respectively, representing positive controls, was mechanically disaggregated and total RNA was isolated using the RNAzolB extraction system (TEL-TEST, TX, USA). No positive control was available for the EWS/FEV fusion gene. RNA was also extracted from a normal mesenchymal tissue to be used as negative control. One microgram of total RNA was reverse-transcribed into cDNA using oligo(dT) primers and reverse transcriptase (Superscript, Gibco-BRL) according to the manufacturer's recommendations.

Control amplification of cDNAs was accomplished by using β-actin specific primers (Adams, 1995) and wild-type EWS primers. The detection of the putative EWS/FLI-1, EWS/ERG junction regions was carried out using primers 11.11 and 22.8 (Zucman et al, 1993) and Erg11 (Delattre et al, 1994) and 22.8 respectively. The PCR reaction consisted of denaturation at 94°C for 30 s, annealing at 68°C for 1 min and elongation at 72°C for 1 min. Thirty-five cycles were performed. The detection of the putative EWS/FEV was carried out using primers EWS22.14 and Fev 11 at the following PCR conditions: 35 cycles consisting of denaturation at 94°C for 30 s, annealing at 65°C for 1 min and elongation at 72°C for 1 min (Peter et al, 1997). The amplification products were analysed on 2% TBE1X-agarose gel.

### Southern blot

DNA was extracted from cases I, II and IV, as well as from two positive controls containing the EWS/FLI-1 transcript, using the Genomix extraction kit (Talent, Trieste, Italy). Ten micrograms of genomic DNA were digested with *Eco*RI, *Hin*dIII, *Bam*HI, *Sac*I and *Xho*l. Restriction fragments were separated by electrophoresis in 0.8% agarose gel O.N. and transferred to nylon membranes (Hybond-N+; Amersham) by standard methods. Filters were hybridized overnight with a ($^{32}$P) radiolabelled probe to a high specific activity by random priming (Prime-it Random Primer Labeling Kit, Stratagene). The EWS probe used was a 593 base pair partial cDNA probe generated by standard PCR. This probe corresponds to nucleotides 720–1313 of the EWS cDNA and includes exons 7–12, which constitute the genomic EWS breakpoint cluster region in pPNETs-ETs (Delattre et al, 1992). The hybridized filters were washed at high stringency and exposed for 2–7 days. Placental DNA was used as normal control and DNAs from two cases with EWS/FLI-I rearrangements were included as positive controls.

*© 1999 Cancer Research Campaign*

### RESULTS

By immunohistochemistry the five ENB cases were negative both with the monoclonal antibody HBA71 and CD99 specific for the protein products of the MIC2 gene.

The cytogenetic analysis of case III resulted in a normal karyotype, and did not reveal numerical and/or structural chromosome abnormalities or the reciprocal t(11;22)(q24;q12), t(21;22)(q22;q12), t(7;22)(p22;q12), t(17;22)(q12;q12) and the t(2;22)(q33;q12) translocations (Rao et al, 1987; Sorensen et al, 1994; Jeon et al, 1995; Kaneko et al, 1996; Peter et al, 1997), or trisomy 8. These results were confirmed by a FISH experiment on metaphase spreads by using a chromosome 22 painting probe which detected two normal chromosome 22 (data not shown).

One hundred interphase nuclei were scored for each case with dual colour FISH using the F7 and 1p3 cosmids flanking the EWS/FLI-1 fusion gene. The absence of t(11;22)(q24;q12) was confirmed in all the three patients analysed: the four signals, two green signals corresponding to F7 probe and two red signals corresponding to 1p3 probe were found apart (Figure 1 A, B). The same experiment performed on a series of Ewing's tumours with a t(11;22), used as positive contol, showed a green/red double spot corresponding to the fusion gene (data not shown).

FISH experiments using a centromeric probe excluded trisomy 8 in cases I and IV, while two different clones were found in case III: one containing three copies of chromosome 8 and the other four (Figure 1 C, D).

RT-PCR analysis using EWS/FLI-1, EWS/ERG and EWS/FEV primers did not detect fusion transcripts in any of the five ENB cases (Figure 2), whereas the positive controls for the first two tranlocations showed the expected specific transcripts. No positive control was available for the EWS/FEV fusion. All the samples showed the expected products when amplified by β-actin specific primers and wild-type EWS primers (Figure 3).

By Southern blot none of the three cases analysed showed any EWS rearrangement after digestion with *Eco*R1, *Hin* dIII, *Sac*I and *Xho*I enzymes, while the two positive controls carrying a t(11;22) and t(21;22) showed a rearranged fragment of 19 and 21 kb and 20 kb respectively, after digestion with *Sac*I and *Xho*I enzymes respectively (Figure 4).

### DISCUSSION

Esthesioneuroblastoma is a malignant tumour characterized by variable histology and conflicting immmunophenotyping, cytogenetic and molecular data. Some authors assert the belonging of ENB to the pPNETs-ETs group of tumours through cytogenetic (Whang-Peng et al, 1987; Cavazzana et al, 1988; VanDevanter et al, 1991) or molecular evidence (Sorensen et al, 1996), whereas others disagree because of opposite immunophenotypic (Nelson et al, 1995) and/or molecular results (Mezzelani et al, 1997; Argani et al, 1998).

In order to address this issue we analysed five consecutive cases of ENB tumour specimens using both cytogenetic and molecular

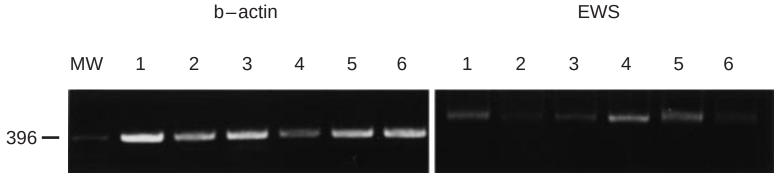

**Figure 3** RT-PCR results. MW: the molecular weight marker is 1 kb. For both β-actin and EWS amplification, samples 1–5 were tumour cDNA from cases I–V, sample 6 was cDNA from normal tissue

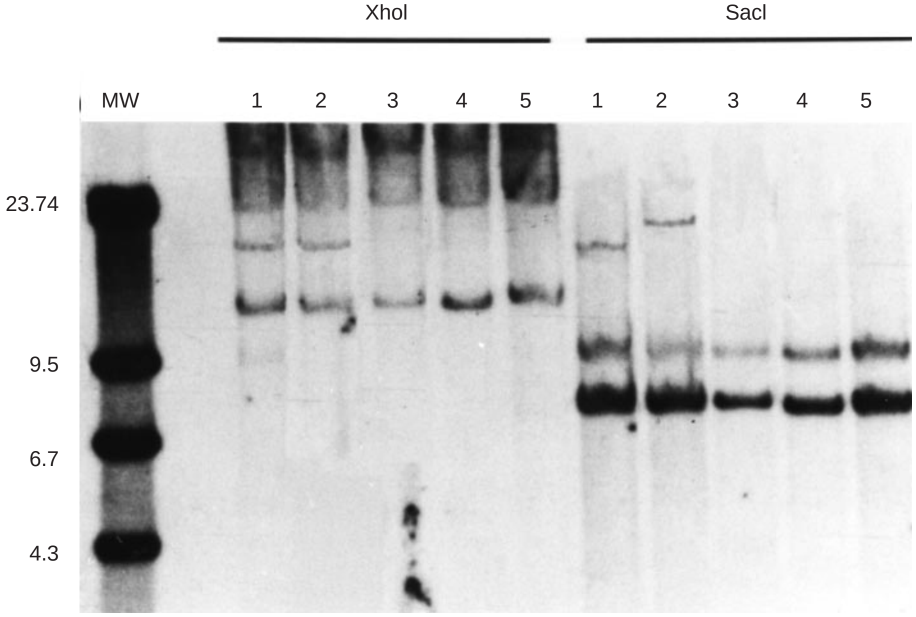

**Figure 4** Southern blot results. MW: the molecular weight marker was λ *Hin*dIII. For both *Xho*I and *Sac*I digestion, samples 1 and 2 were the two positive controls and samples 3–5 were case I, II and IV respectively

© 1999 Cancer Research Campaign techniques. All cases were consistent with olfactory neuroblastoma in terms of morphology and tumour-specific immunophenotype, as well as development site and clinical presentation.

As for MIC2 gene expression, all our cases showed null MIC2 gene immunophenotype, in agreement with the recent report of Nelson et al (1995). Furthermore, the results we obtained on the original uncultured tumour specimens by dual colour FISH in cases I, III and IV, RT-PCR in all cases, and by Southern blot in cases I, II and IV all confirmed the absence of any EWS rearrangements. In fact we did not find any evidence of EWS/FLI-1 fusion gene by FISH on interphase nuclei, or of EWS/FLI-1 fusion transcripts, or of EWS/ERG and EWS/FEV variants by cDNA amplification. The same was true for Southern blot analysis that confirmed the EWS gene integrity. Case III was the only tumour whose cells were successfully cultured in vitro short-term culture. The culture was harvested after 10 days, and the metaphases showed a normal 46,XY karyotype. Since it has been reported that during the in vitro culture fibroblasts could overgrow tumour cells (Jin et al, 1995), we must take this potential bias into account.

We attribute the robustness of our approach to three factors. First, the reliability of the RT-PCR results are based on the fact that the cDNA adequacy was tested by both β-actin and EWS amplification as well as on the amplification of positive controls. Second, even though in theory, rearrangements of the EWS locus could be excluded only by complete sequencing of the tumour DNA, we undertook an extended Southern blot analysis. In our experience, pPNETs-ETs DNA carrying EWS/FLI-1 transcript does not always show EWS rearrangement when digested by *Eco*R1 and *Hin*dIII enzymes. In fact, only the use of rare cuttering enzymes, such as *Sac*I and *Xho*I, allowed us to demonstrate the presence of EWS rearrangements in these samples and thus to ultimately exclude the presence of rearrangements in our ENB cases. Third, FISH allowed a cell by cell analysis that excluded the presence of the EWS/FLI-1 transcript in the three tested ENB and, by contrast, showed it in a series of pPNETs-ETs.

The presence, in Case III, of two different clones containing three and four copies of chromosome 8, respectively, is not sufficiently understood. Although, trisomy 8 is described as the most common secondary anomaly in neuroectodermal tumours (Heim and Mitelman, 1995), it is also a frequent abnormality in different tumour types. In fact, trisomy 8 is a non-random secondary change in other soft tissue tumours, such as clear cell sarcoma (Travis et al, 1992) and myxoid liposarcoma (Sreekantaiah et al, 1994) and appears as the only chromosome abnormality in several myeloid and lymphoid disorders (Mitelman, 1985; Haim and Mitelman, 1995) along with superficial and deep fibromatosis (Somers et al, 1987; Dal Cin et al, 1994).

In conclusion, on the basis of our data and a critical analysis of the literature, we are inclined to assert that ENB may represent a distinct entity as opposed to being a member of the pPNETs-ETs. In fact, only four out of 27 ENB cases (Wang-Peng et al, 1987; Cavazzana et al, 1988; Sorensen et al, 1996) cytogenetically or molecularly analysed, showed the EWS/FLI-1 fusion gene. As already argued (Nelson et al, 1995) two of the three ENB cell lines described to carry the t(11;22)(q24;q12)-derived EWS/FLI-1 fusion transcript (Whang-Peng et al, 1987; Cavazzana et al, 1988; Sorensen et al, 1996) probably represent metastatic lesions deriving from a primary pPNET-ET in the chest wall and in the paraspinal region, respectively, and metastasizing to the nasal cavity. Two additional primary ENBs described by Sorensen et al (1996) could represent pPNETS-ETs or extraskeletal ETs arising in the nasal fossa, as previously described by Pontius and Sebek (1982), and Lane and Ironside (1990). The remaining three cases with abnormal karyotype described by other authors did not show a t(11;22) translocation (Castaneda et al, 1991; Van Devanter et al, 1991; Jin et al, 1995). Finally, the immunophenotypic data from 43 cases were all consistent with a lack of MIC2 protein product (Nelson et al, 1995; Argani et al, 1998).

### ACKNOWLEDGEMENTS

This work was supported by grants from AIRC/FIRC (Associazione and Fondazione Italiana per la Ricerca sul Cancro).

### REFERENCES

Adams V, Kempf W, Hassam S and Briner J (1995) Determination of hexokinase isoenzyme I and II composition by RT-PCR: increased hexokinase isoenzyme II in human renal cell carcinoma. *Biochem Mol Med* **54**: 53–58

Argani P, Perez-Ordonez B, Xiao H, Caruana SM, Huvos AG and Ladanyi M (1998) Olfactory neuroblastoma is not related to the Ewing family of tumors absence at EWS/FLI1 gene fusion and MIC2 expression. *Am J Surg Pathol* **22**: 391–398

Banerjee AK, Sharma BS, Vashista RK and Kak VK (1992) Intracranial olfactory neuroblastoma: evidence for olfactory epithelial origin. *J Clin Pathol* **45**: 299–302

Burger PC and Scheithauer BW (1993) *Atlas of Tumor Pathology. Tumors of the Central Nervous System*, pp. 200–202. Washington: Armed Forces Institute of Pathology

Cattoretti G, Beker MHG, Key G, Duchrow M, Schluter C, Galle J and Gerdes J (1992) Monoclonal antibodies against recombinant parts of the Ki-67 antigen (MIB 1 and MIB 3) detect proliferating cells in microwave processed formalin-fixed praffin sections. *J Pathol* **168**: 357–363

Cavazzana AO, Navarro S, Noguera R, Reynolds PC and Triche TJ (1988) Olfactory neuroblastoma is not a neuroblastoma but is related to primitive neuroectodermal tumor (PNET). *Prog Clin Biol Res* **271**: 463–473

Chan JCK, Tsang WJW, Sereviratne S and Pau MY (1995) The MIC2 antibody 013 practical application for the study of thymic epithelial tumors. *Am J Surg Pathol* **19**: 1115–1123

Dal Cin P, Sciot R, Aly MS, Delabie J, Stas M, De Wever I, Van Damne B and Van Den Berghe H (1994) Some desmoid tumors are characterized by trisomy 8. *Genes Chromasomes Cancer* **10**: 131–135

Delattre O, Zucman J, Plougastel B, Desmaze C, Melot T, Peter M, Kovar H, Joubert I, De Jong P, Rouleau G, Aurias A and Thomas G (1992) Gene fusion with and ETS DNA-binding domain caused by chromosome translocation in human tumors. *Nature* **359**: 162–165

Delattre O, Zucman J, Melot T, Sastre Garau X, Zucker JM, Lenoir GM, Ambros PF, Sheer D, Turc-Carel C, Triche TJ, Aurias A and Thomas G (1994) The Ewing family of tumors. A subgroup of small-round cell tumors defined by chimeric transcript. *N Eng J Med* **331**: 294–299

Desmaze C, Zucman J, Delattre O, Melot T, Thomas G and Aurias A (1994) Interphase molecular cytogenetics of Ewing's sarcoma and peripheral neuroepithelioma t (11;22) with flanking and overlapping cosmid probes. *Cancer Genet Cytogenet* **74**: 13–18

Frierson HF, Ross GW, Mills SE and Frankfurter A (1990) Olfactory neuroblastoma. Additional immunohistochemical characterization. *Am J Clin Pathol* **94**: 547–553

Hasle H, Clausen N, Pedersen B and Bendix-Hansen K (1995) Myelodysplastic syndrome in a child with constitutional trisomy 8 mosaicism and normal phenotype. *Cancer Genet Cytogenet* **79**: 79–81

Heim S and Mitelman F (1995) Cancer cytogenetics chromomomal and molecular genetic aberrations of tumor cells. pp 494–495. New York: Alan R. Liss, Inc.,

Hyams VJ, Batsakis JG and Michaels LE (1998) *Atlas of Tumor Pathology. Tumors of the Upper Respiratory Tract*, 2nd series, fascicle 25, pp. 239–248. Washington, DC, Armed Forces Institute of Pathology

Jeon IS, Davis JN, Braun BS, Sublett JE, Roussel MF, Denny CT and Shapiro DN (1995) A variant Ewing's sarcoma translocation (7;22) fuses the EWS gene to the ETS gene ETV1. *Oncogene* **10**: 1229–1234

© 1999 Cancer Research Campaign

Jin Y, Mertens F, Arheden K, Mandhal N, Wennerberg J, Dictor M, Heim S and Mitelman F (1995) Karyotypic features of malignant tumors of the nasal cavity and paranasal sinuses. *Int J Cancer* **60**: 637–641

Kadish S, Goodman M and Wangt CC (1976) Olfactory neuroblastoma. *Cancer* **37**: 1571–???

Kaneko Y, Yoshida K, Handa M et al (1996) Fusion of an ETS-family gene, EIAF, to EWS by t(17;22) (q12:q12) chromosome translocation in an undifferentiated sarcoma of infancy. *Genes Chrom Cancer* **15**: 115–121

Lane S and Ironside JW (1990) Extra-skeletal Ewing's sarcoma of the nasal fossa. *Laryngol Otol* **104**: 570–573

Lichter P, Change Tang C, Call K, Hermanson G, Evans GA, Housman D and Ward DC (1990) high-resolution mapping of human chromosome 11 by in situ hybridization with cosmid clones. *Science* **247**: 64–67

Mark HFL (1996) Constitutional trisomy 8 mosaicism and cancer. *Cancer Genet Cytogenet* **86**: 87–88

Mezzelani A, Tornielli S, Sard L, Radice MT, Minoletti F, Pierotti MA and Pilotti S (1997) Is esthesioneuroblastoma a member of the primitive peripheral neuroectodermal tumors? *Cytogenet Cell Genet* **77**: 145

Mitelman F (1985) *Human Chromosome Abnormalities: Catalogues and Collections*, pp. 203–248. Alan R. Liss, Inc, New York

Nelson RS, Perlman EJ and Askin FB (1995) Is esthesioneuroblastoma a peripheral neuroectodermal tumor? *Hum Pathol* **26**: 639–641

Peter M, Couturier J, Pacquement H, Michon J, Thomas G, Magdelenat H and Delattre O (1997) A new member of the ETS family fused to EWS in Ewing tumors. *Oncogene* **14**: 1159–1164

Pontius K and Sebek B (1982) Extraskeletal Ewing's sarcoma of the nasal fossa. *Am J Clin Pathol* **75**: 410–415

Rao VN, Papas TS and Reddy ES (1987) Erg, a human ets-related gene on chromosome 21: alternative splicing, polyadenylation, and translation. *Science* **237**: 635–639

Ried T, Baldini A, Rand TC and Ward DC (1992) Simultaneous visualization of seven different probes by in situ hybridization using combinatorial fluorescence and digital imaging microscopy. *Proc Natl Acad Sci USA* **89**: 1388–1392

Shanmugaratnam K (1991) World Health Organization. *International Histological Classification of Tumours. Histological Typing of Tumours of the Upper Respiratory Tract and Ear*. Springer-Verlag, Berlin

Somers KD, Winters BA, Dawson DM, Leffell MS, Wright GL, Devine CJ, Gilbert DA and Horton CE (1987) Chromosome abnormalities in Peyronie's disease. *J Urol* **37**: 672–675

Sorensen PHB, Lessnick SL, Lopez-Terrada D, Liu XF, Triche TJ and Denny CT (1994) A second Ewing's sarcoma translocation, t (21;22), fuses the EWS gene to another ETS-family transcription factor, ERG. *Nature Genetics* **6**: 146–151

Sorensen PHB, Wu JK, Berean KW, Lim JF, Donn W, Frierson HF, Reynolds LP, Lopez-Terrada D and Trichet J (1996) Olfactory neuroblastoma is a peripheral primitive neuroectodermal tumor related to Ewing sarcoma. *Proc Natl Acad Sci USA* **93**: 1038–1043

Sreekantaiah C, Ladanyi M, Rodriguez E and Chaganti RSK (1994) Chromosomal aberrations in soft tissue tumors. Relevance to diagnosis, classification, and molecular mechanisms. *Am J Pathol* **144**: 1121–1133

Travis JA and Bridge JA (1992) Significance of both numerical and structural chromosomal abnormalities in clear cell sarcoma. *Cancer Genet Cytogenet* **64**: 104–106

VanDevanter DR, George D, McNutt MA, Vogel A and Luthardt F (1991) Trisomy 8 in primary esthesioneuroblastoma. *Cancer Genet Cytogenet* **57**: 133–136

Whang-Peng J, Freter CE, Knutsen T, Nanfro JJ and Gazdar A (1987) Translocation t (11;22) in esthesioneuroblastoma. *Cancer Genet Cytogenet* **29**: 155–157

Wood GS and Warnke R (1981) Suppression of endogeneous avidin binding activity in tissues and its relevance to biotin–avidin detection systems. *J Histochem Cytochem* **29**: 1196–1204

Zucman J, Delattre O, Desmaze C, Plougastel B, Joubert I, Melot T, Peter M, De Jong P, Rouleau G, Aurias A and Thomas G (1992) Cloning and characterization of the Ewing's sarcoma and peripheral neuroepithelioma t (11;22) translocation breakpoint. *Genes Chrom Cancer* **5**: 271–277

Zucman J, Melot T, Desmaze C, Ghysdael J, Plougastel B, Peter M, Zucker JM, Triche TJ, Sheer D, Turc-Carel C, Ambros P, Combaret V, Lenoir G, Aurias A, Thomas G and Delattre O (1993) Combinatorial generation of variable fusion proteins in the Ewing family of tumors. *EMBO J* **12**: 4481–4487

© 1999 Cancer Research Campaign
